# Clinical Outcomes and Resource Efficiency of a Telehealth Model for New Lower Gastrointestinal Bleeding Referrals: A Tertiary Colorectal Outpatient Service Audit

**DOI:** 10.1155/ijta/9945160

**Published:** 2025-01-09

**Authors:** Zainab Naseem, Qamar M. Butt, Amina Shaukat, Rachael Grenfell-Dexter, Junzhe Zhu, Basil D'Souza, Neil Strugnell

**Affiliations:** ^1^Department of Medicine and Health, University of Sydney, Sydney, Australia; ^2^Department of Colorectal Surgery, Northern Hospital Epping, Epping, Australia; ^3^Department of Surgery, University of Melbourne, Melbourne, Australia

**Keywords:** colorectal clinic, COVID, lower gastrointestinal bleeding, PR bleeding, proctology, telehealth, telephonic consult

## Abstract

**Introduction:** Amidst the COVID-19 pandemic, telehealth (TH) has gained increasing relevance in colorectal patient care, prompting an audit of the TH clinic at a tertiary colorectal unit. This study evaluated telephone-based consultations' clinical efficacy and diagnostic thresholds for new lower gastrointestinal (LGI) bleeding referrals.

**Methods:** We conducted a quality assurance audit of the per rectal (PR) bleeding TH clinic at Northern Hospital Victoria, evaluating new LGI bleeding referrals. Data from July 2021 to June 2023 were extracted from the Referral Management System (RMS) and analyzed. The study focused on newly referred patients, excluding those previously seen or awaiting procedures. Clinical efficacy was determined through sensitivity, specificity, and predictive values, with a receiver operating characteristic (ROC) curve assessing the TH method's discrimination threshold.

**Results:** Of the 239 patients, 131 met the inclusion criteria, with a compliance rate of 96%. The TH clinic demonstrated a sensitivity of 75.76% and specificity of 52.46% in distinguishing between colorectal and anal pathologies. The average time from referral to a diagnostic procedure was 9.75 weeks, with a reduction in median wait time for first appointments from 19 weeks prepandemic to 3.6 weeks. A cost–benefit analysis highlighted TH clinics' ability to reduce overhead costs and maintain a revenue stream despite reduced face-to-face consultations.

**Conclusion:** Our study concludes that the telecare service model serves as an effective complementary approach for managing new LGI bleeding referrals. Further research on long-term outcomes and cost-effectiveness is necessary to fully assess telecare as a potentially sustainable hybrid model.

## 1. Introduction

Since the COVID-19 pandemic, telehealth (TH) has emerged as a potential alternative modality for providing health care to patients with surgical diseases. This is equally true for the colorectal (CR) patient cohort.

The crisis response triggered by this pandemic has resulted in a 65% decrease in outpatient attendance with a 90% drop in endoscopic procedures in CR cancer patients noted in the early phase of the pandemic [1]. Due to the imperative for physical distancing and travel restrictions, the proportion of patients presenting with advanced CR cancer has increased in many international centers [2]. A review of the Binational Colorectal Cancer Audit (BCCA) registry of Australia and New Zealand also demonstrated an increased number of urgent and emergent procedures performed due to delayed presentation of CR cancers (Klionsky et al.). Moreover, the lockdowns and distancing requirements have also impacted the delivery of outpatient services to those with benign CR conditions [3].

TH has been utilized to provide primary and specialised medical care to rural and regional areas of Australia, both before and during the COVID pandemic. Before the pandemic, TH/telemedicine facilities were being recognised for their ease of use in multidisciplinary care, providing posttreatment consultations with patients in remote areas, stoma care, and family support with screening of familial cancer and maintaining a more compliant follow-up for ongoing holistic care [4–6]. The body of literature concerning this modality's feasibility and its ongoing use in the comprehensive care of CR cancer patients is now growing. However, its effectiveness in handling new referrals for a variety of CR conditions remains unknown. The rapid adoption of TH by providers and its acceptance among patients necessitate a review of its quality and clinical outcomes. We present the results of the quality assurance assessment of the per rectal (PR) bleeding TH clinic, an initiative by Northern Health Victoria. This audit objectively evaluates the clinic's performance and role as a sustainable ongoing service.

## 2. TH Clinic Setup

In response to the challenges presented by the COVID-19 pandemic, our metropolitan CR service adopted a TH platform designed explicitly for CR care for outpatient services. The initiative was coordinated alongside the establishment of a virtual emergency department to reduce in-person visits and the risk of viral transmission. One aspect of this platform was a telephone-based clinic attending to new patients referred for management of lower gastrointestinal (LGI) bleeding. This PR bleeding clinic was not merely a temporary measure; it has since evolved into a more permanent feature of our healthcare delivery model. Initially, CR surgeons were reluctant to adopt this method of consultation. However, it soon became evident that there was a strong state-wide and local institutional policy directive requiring all clinics, across both medical and surgical specialties, to adopt this consultation method, as an adjunct to the limited availability of in-person consultations, during the COVID-19 pandemic. Even before the COVID pandemic, due to the growth of the catchment population, meeting the service requirements for in-person assessments of patients referred for overt PR bleeding (referral clinical code 5094-LGI bleeding) had become increasingly challenging. As a result, we decided to assess the clinical safety, efficiency, and timeliness of PR bleeding assessments both before and after the introduction of this specific service. The first consultant-run, telephone-based PR bleeding clinic was held in July 2021 and has since expanded. Starting in February 2023, the TH platform has been maintained by an additional fellow clinic.

The rationale for conducting this service audit was to scrutinise the clinical efficacy and cost-effectiveness of utilizing a TH approach in the management of patients with LGI bleeding. We aimed to gauge not only the immediate benefits but also the long-term applicability of such a system.

## 3. Material/Patients and Methods

### 3.1. Institutional Approval

The Department of Ethics and Governance at the Northern Centre for Health Education Research (NCHER) approved this project.

### 3.2. Data Collection

Data for this study was extracted from the Referral Management System (RMS), a component of the Clinical Patient Folder (CPF). The CPF is a digital repository employed across all Northern Health campuses to store and retrieve clinical documents. This system ensures digital access to patient medical records. The RMS particularly catalogues all specialist clinic referrals, capturing essential details such as patient demographics, referral source, priority, and the mode, type, and status of the TH clinic appointment. Data were collected from July 1, 2021, to June 30, 2023.

### 3.3. Inclusion and Exclusion Criteria

All newly referred patients with the presenting complaint of overt LGI bleeding with or without associated occult bleeding were included. Patients with isolated occult bleeding were not analyzed as in our hospital these referrals are streamed through to a gastroenterology physician and nurse clinic. Patients discharged from the CR surgery unit or previously seen in specialist clinics were excluded from the study. New referrals who were reviewed via telephonic consultations who had already been assessed through other channels and who were already awaiting diagnostic endoscopic procedures were also excluded from the study.

### 3.4. Data Variables

The audit data include several categories of variables, detailed as follows:

1. Referral data: This category captures the total number of patients triaged for teleconsultation, attendance details, and referral sources to establish service performance indicators.
2. Demographic data: Variables such as age, gender, and date of attendance were retrieved from the RMS.
3. Clinical data: Information related to telephone consultations was extracted from digital records in the CPF. Key clinical variables included “red flags” such as anaemia, weight loss, tenesmus, family history of CR cancer, and personal history of related pathology. Data concerning patients who later had face-to-face consultations and the reasons for these consultations were also collected.
4. Procedural data: This category included the type of procedure (diagnostic or definitive surgical) and the associated waiting times.

## 4. Study Outcomes

The primary objective was to assess the efficacy of telephone consults in distinguishing patients with colonic or rectal pathologies inclusive of the possibility of sinister diagnosis (CR category) from the ones who may have benign anal conditions (anal category).

The secondary objectives were to evaluate the mean time from referral to the management outcome, waiting time from referral registration to first appointment, failure to attend (FTA) the first appointment, rate of face-to-face consultations, and factors affecting this rate.

## 5. Statistical Analysis

Descriptive statistical methods were used to analyse demographic and clinical data. Patients were sorted into two diagnostic groups based on clinical impressions: Those with colonic pathologies such as diverticular disease, colonic polyps, inflammatory bowel disease, and CR cancer were placed in the “CR pathology” group, while those with conditions like haemorrhoids and fissures were classified into the “anal pathology” group. The proportions and frequencies for each group were calculated. Sensitivity, specificity, and positive and negative predictive values were used to evaluate the clinical efficacy of TH in detecting CR pathology. A receiver operating characteristic (ROC) curve was plotted to assess the discrimination threshold of the TH method, using the final diagnoses from scheduled procedures as the reference standard. The average time (weeks) from referral to a Category 1 procedure was calculated. Lastly, the need for in-person consultations was quantified as a percentage, and a logistic regression analysis was conducted to identify factors predicting the necessity for face-to-face consultations.

## 6. Results

**Figure 1:**
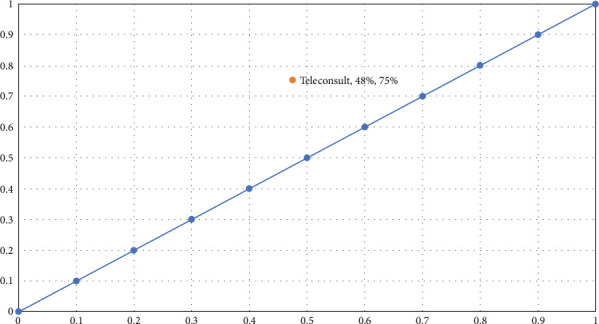
Receiver operating characteristic (ROC) analysis indicating the diagnostic threshold for colorectal pathology.

Two hundred and thirty-nine patients' data were collected from the RMS, with 131 meeting the inclusion criteria. Out of the 239 patients, 14 had an initial TH consultation but did not proceed with any diagnostic procedure due to choosing another healthcare provider or declining. Patients with previous clinic reviews or waiting for scheduled procedures were excluded from the study. The compliance rate was 96% (126/131), with a mean age of 53.61 years and a male-to-female ratio of 1.17:1. Anaemia was the most common associated symptom (49%), among other red flag indicators and risk factors such as personal or family history of CR cancer (Table 1). Of the attendees, 78 (61.9%) were thought likely to have the possibility of a significant colonic pathology inclusive of the possibility of sinister rectal pathology (CR diagnostic group). As a result of the diagnostic procedure, it was determined that 66 patients (84.6%) had a colonic pathology (Table 2). Sensitivity was calculated as the proportion of true positives (patients correctly identified as having the disease: 50) among all patients who actually had the disease (true positives and false negatives: 50 + 16), yielding a sensitivity of 75.76%. Specificity was calculated as the proportion of true negatives (patients correctly identified as not having the disease) among all patients who did not have the disease (true negatives and false positives: 32 + 29), yielding a specificity of 52.46%. The ROC curve area depicts a trade-off between sensitivity and specificity (Figure 1) for a diagnostic test. The closer the curve follows the left-hand border and then the top border of the ROC space, the more accurate the test. Conversely, the closer the curve comes to the 45° diagonal of the ROC space, the less accurate the test. The point marked on the curve shows a specific balance between sensitivity and specificity chosen for the TH consultations in this particular study. A ROC curve analysis indicated a moderate discrimination threshold for the telephone consultation to differentiate a predominate colonic pathology.

Fourteen patients (11.1%) were advised to undergo in-person consultations, with a mean age of 51.5 years (compared to 53.8 years in the telephone consultation-only group). This suggests an increased likelihood of alternative anal pathology diagnoses in this younger cohort. In a subgroup analysis, colonoscopy (51%) was the primary diagnostic procedure for patients 40 years and older. Forty-four percent of the younger cohort (< 40 years) also underwent colonoscopy rather than sigmoidoscopy, due to the lack of opportunity to perform physical examination at the time of initial telephone assessment (Table 3). However, logistic regression analysis found no significant correlations between the likelihood of needing an in-person consultation and variables such as age, gender, or red flag indicators. The mean time from referral to any diagnostic procedure was 9.75 weeks.

The median wait time for a first appointment postreferral decreased from 19 weeks in 2019–2020 to 3.6 weeks in 2021–2022, with a slight rise to 4.0 weeks in 2022–2023. For patients FTA their initial appointment, the rate improved from 16.1% (193 out of 1199) in 2021–2022 to 12.5% (166 out of 1330) in 2022–2023 (Tables 4 and 5).

## 7. Discussion

Healthcare delivery has undoubtedly changed since the pandemic hit. The widespread use of telecare has paved the way for its routine inclusion in daily clinical practice. Its global adoption and acceptability in the CR field are also noteworthy. Data from Kaiser Permanente Northern California Centre indicated the exponential use of telemedicine in the CR speciality (5.7%–62.2%) along with gynaecology and trauma service after the pandemic [7]. The ease and comfort with which patients have adapted to telecare raises questions about its optimal applications in the future [8, 9]. Accordingly, this project is aimed at assessing past effectiveness to gauge its potential as a sustainable healthcare delivery method. Our study shows that high compliance rates in telephonic consultations indicate increased patient adaptability despite the demographic and ethnic diversity served by our institution. This has turned the service into a resource-efficient healthcare delivery method [10].

A Proctology Algorithm Working Group study reported a 68% compliance rate among patients, with 30.4% of nondeferrable cases undergoing procedures during the COVID-19 pandemic [11]. A group of 47 Italian proctology experts considered clinical practice guidelines for telemedicine during global lockdowns. While there was consensus on the risks of misdiagnosis, particularly of cancer, the technology was deemed appropriate for managing various proctological conditions to reduce waiting times [12]. Despite the limitations of TH in proctology, there is room for this service to streamline the workflow in busy CR clinics.

Our study found a sensitivity of 75.76% and a specificity of 52.46% for predicting CR pathologies as the cause of LGI issues. However, these figures and the ROC curve indicate a moderate triaging threshold. The ROC curve also suggests that achieving higher sensitivity would require more patients, significantly younger and low-risk individuals, to undergo unnecessary invasive procedures like colonoscopy. Although a standardised questionnaire was not employed, the consultations were structured to include pertinent clinical details, including a thorough clinical impression and a management plan, all documented according to the CPF consult note format. This structured format ensured that critical elements relevant to distinguishing between CR and anal pathologies were consistently captured.

The early pandemic period saw higher waiting times despite reduced referrals due to operational constraints and deferred care; the subsequent introduction of TH enabled us to manage an increased backlog of referrals effectively, resulting in reduced waiting times. The transition to TH proved crucial in enhancing service delivery and maintaining continuity of care during and after the pandemic.

A cost–benefit analysis revealed significant financial advantages of CR TH clinic. Traditional face-to-face clinics necessitate overhead costs, including secretarial support, which can amount to approximately AUS$30 per hour for a 3.5-h session in our institution. While our initial analysis focused on the model of care-related savings from administration/clerical costs, it is essential to augment this by acknowledging the substantial cost savings associated with nursing services in the TH setting. According to the new enterprise agreement, the morning and afternoon shift allowance for nursing staff is AUS$32.50 [13]. Incorporating these specific allowances allows us to accurately evaluate the economic benefits associated with the TH clinic. Additionally, the initial TH setup cost, as supported by the Victorian Health Department, was approximately AUS$4000, which includes the purchase of necessary technology, software, and training for staff. This one-time cost is quickly offset by the ongoing savings in reduced physical space requirements and the elimination of on-site administrative staff during consultations. TH clinics can operate with reduced overhead, eliminating the need for physical space and on-site administrative staff during consultations. Moreover, these clinics offer the unique benefit of maintaining a revenue stream even when in-person consultations are not feasible. By enabling consultations that would otherwise be deferred, telephone clinics ensure continuity of care and business operations, which is particularly crucial in situations where face-to-face interactions are limited. This adaptability not only sustains the clinic's financial health but also reduces patient backlog and enhances overall healthcare efficiency, contributing to a more cost-effective healthcare delivery model.

In the context of assessing the expenses associated with providing colonoscopy and flexible sigmoidoscopy services, a critical assumption is made concerning the equivalence of downstream costs between TH and face-to-face consultations. However, it is essential to underscore the uncertainty surrounding the actual parity of these costs. The unique dynamics of TH introduce a nuanced consideration—the potential for a slightly higher investigation-related cost. This arises from the circumstance where patients with positive faecal occult blood, in whom overt PR bleeding is absent, may opt not to attend a traditional face-to-face consultation. In contrast, these individuals may still proceed with colonoscopic or flexible sigmoidoscopic evaluation if they have already been scheduled for the procedure through the TH clinic. Recognizing this plausible scenario is paramount, as it serves to demonstrate our awareness of the intricacies involved in the comparative costing of these healthcare delivery modalities.

## 8. Limitations of the Study

We recognise that, during the pandemic, patients in metropolitan Melbourne were generally averse to undergoing investigations due to social distancing and lockdown restrictions. For this reason, patients who declined investigations or endoscopic procedures were not included in this dataset. Although we do not have specific data, we have no reason to assume otherwise. Our study did not include data on patients who did not proceed with expedited procedures following TH consultations, as the focus was specifically on those requiring diagnostic procedures for new LGI bleeding referrals. Normally, these patients would be discharged from the clinic, making it difficult to collect accurate data.

Those referrals which involve positive faecal occult blood but lack overt PR bleeding are, as per the institutional triaging agreement, redirected to the gastroenterology clinics. Through the application of TH, these patients undergo the consent process to be included in the colonoscopy waiting list. It is essential to note that within this referral cohort, there exists a spectrum of diagnoses, including cases of CR cancer. While the quantification of such cases has been addressed elsewhere, it is crucial to emphasize that our study specifically focuses on CR referrals originating from general practitioners—particularly those patients presenting with overt PR bleeding who are referred to CR surgical clinics.

## 9. Blueprint for the Future

We suggest a multifaceted strategy for the long-term implementation of telemedicine in handling LGI bleeding referrals. This calls for a thorough cost–benefit analysis, the formulation of standardised consultation protocols, and multidisciplinary cooperation. One of the suggested standardised protocols is avoidance of duplication and streamlining of rectal bleeding diagnosis (ADSORB) guidelines which focus on the “avoidance of duplication and streamlining of rectal bleeding diagnosis”. This way can substantially elevate the efficiency and effectiveness of TH clinics dealing with such referrals. Given the rapid evolution of direct-to-consumer telemedicine services, the streamlined approach proposed by these guidelines can be especially beneficial in addressing CR concerns [14]. This would facilitate better triaging and help identify when an in-person consultation is necessary for proctological conditions that may not require extensive endoscopic diagnostic assessments. A long-term reimbursement strategy is worthwhile investigating, given the ongoing usage. A pilot program may be a wise first step in establishing key performance indicators for continuing quality assurance. While at this stage, we endorse telecare as a complementary outpatient service for new LGI bleeding referrals, and further research focusing on long-term outcomes and cost-effectiveness will be required to fully elucidate its potential role as a substitutive method of care.

## Figures and Tables

**Table 1:** Characteristics of the study population (demographic and clinical).

Extracted electronic referrals	239
Study population	126
Age (mean, range)	53.61 years (18–86)
*Gender*	*n* (%)
Male	68 (54%)
Female	58 (46%)
*Clinical impression*	
Predominate colorectal pathology	78 (61.9)
Predominate anal pathology	48 (38.1)
*Red flags and risk factor distribution*	
Anaemia	49.2%
Family history	13.5%
Altered bowel habits	10.3%
Unexplained weight loss	0.8%

**Table 2:** Postdiagnostic evaluation: Distribution of colonic and proctology pathologies.

Colorectal pathologies	IBD	3
	Diverticular disease	18
	Adenoma	20
	Advanced adenoma	17 (10 (size > 10 mm), 7 (villous component))
	Sessile serrate lesion/adenoma (SSP/SSA)	7
	Colorectal cancer	0
	Solitary rectal ulcer	1

Anal pathologies	Hemorrhoidal disease	44
	Fissure	2
	Fistula in ano	1

**Table 3:** Subgroup procedural analysis.

**Age subgroups (years)**	**Procedure type**			**Total**
	**Colonoscopy**	**Flexi sigmoidoscopy**	**Surgical procedures**	
< 40	12	14	1	27
40–50	25	1	0	26
> 50	67	4	2	73
Total	104	19	3	126

**Table 4:** Waiting time for first appointment.

**Median number of weeks from referral registered to first appointment**			
	**2019–2020**	**2021–2022**	**2022–2023**
Median week	19	3.6	4.0

**Table 5:** Rate of failure to attend the first appointment.

**Failed to attend (FTA) first appointment after referral**		
	**2021–2022**	**2022–2023**
FTA	193	166
Total	1199	1330
% FTA	16.1%	12.5%

## Data Availability

The data is a property of the Department of Medical Information, Northern Hospital, Victoria, and can be accessed on reasonable request made to the corresponding author.
